# Chemical Annealing Restructures RNA for Nanopore Detection

**DOI:** 10.1021/jacs.4c03753

**Published:** 2024-05-01

**Authors:** Casey M. Platnich, Max K. Earle, Ulrich F. Keyser

**Affiliations:** Cavendish Laboratory, University of Cambridge, Cambridge CB3 0HE, United Kingdom

## Abstract

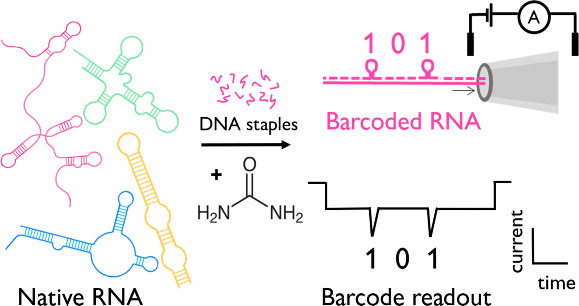

RNA is a key biochemical marker, yet its chemical instability and complex secondary structure hamper its integration into DNA nanotechnology-based sensing platforms. Relying on the denaturation of the native RNA structure using urea, we show that restructured DNA/RNA hybrids can readily be prepared at room temperature. Using solid-state nanopore sensing, we demonstrate that the structures of our DNA/RNA hybrids conform to the design at the single-molecule level. Employing this chemical annealing procedure, we mitigate RNA self-cleavage, enabling the direct detection of restructured RNA molecules for biosensing applications.

The economic, social, and medical impacts of viral outbreaks have proven devastating in recent years, underlining the immediate need for rapid and adaptable biosensing technologies. RNA is a potent biomarker for sensing disease at all stages, typically identified within biological samples via reverse transcription quantitative polymerase chain reaction.^1^ The necessary reverse transcription and subsequent amplification steps introduce enzymatic biases, complicating the relative quantification of RNAs in complex samples.^2^ Moreover, these techniques lack single-molecule sensitivity, meaning that low abundance species may go undetected due to ensemble averaging.^3^

To simplify the identification and quantification of nucleic acids, single-molecule nanopore sensing is a promising platform.^4^ A quartz glass nanopore (5–10 nm in diameter) is placed between two reservoirs containing an aqueous ionic solution and the analyte, while the ionic current passing through the pore is measured. Applying a voltage across the nanopore results in translocation of the analyte and thus a temporary pore obstruction, producing a measurable current drop. The characteristics of this current signal provide information about the analyte’s length, shape, and structure.^5^

Leveraging the principles of DNA nanotechnology,^4^ it is possible to design three-dimensional “barcodes” using short complementary oligonucleotides, which self-assemble onto DNA or RNA sequences of interest.^6−8^ Each target sequence is unique and thus corresponds to a specific barcode with a distinct current signature, enabling multiplexed, single-molecule detection of nucleic acids with no enzymatic amplification required.^7^

The yield of the correctly formed duplex barcode is impacted by the inherent secondary structure of the nucleic acid target.^9^ While DNA primarily exists in the canonical double helix, RNA may sample a plethora of stable conformational isoforms, including hairpin loops, multibranch loops, and kissing loops.^10^ To incorporate RNA into barcoded DNA nanostructures for direct detection, native secondary structures must be disrupted to promote RNA/DNA base pairing. In DNA nanotechnology, thermal annealing is employed to melt the double helix: RNA/DNA are heated (often to 95 °C) and then slowly cooled, enabling equilibration at each temperature.^11^ In doing so, the mixture is brought to the thermodynamic equilibrium state, corresponding to the desired design. This methodology is suitable for DNA structures but limits the yield of RNA barcodes because RNA undergoes self-cleavage at elevated temperatures.^12,13^ As such, innovative procedures to generate RNA/DNA hybrids while minimizing the RNA degradation are required.

We present a new protocol which uses chemical (as opposed to thermal) denaturation of RNA sequences, facilitating the binding of complementary DNA staples to reshape the RNA. Specifically, we employ urea, which has previously been shown to linearly decrease the melting temperature of DNA duplexes with increasing concentration (2.25% per molar).^14^ Urea disrupts Watson–Crick base pairing through its favorable, nonspecific interactions with the “amide-like” surfaces of the nucleobases.^15^ Here, we use these interactions to break up RNA’s native secondary structure. In the presence of a 5-fold stoichiometric excess of DNA staples (30–50 nt in length, detailed in the Supporting Information), the urea allows the system to reach the designed thermodynamic minimum. The result is the formation of hybrid duplexes at *T* = 25 °C.

Barcoded species produced by using chemical annealing are stable in the presence of urea, as evidenced by agarose gel electrophoresis (AGE) and solid-state nanopore measurements. We find that by impeding the self-folding of RNA through the hybridization of the DNA complements hydrolysis is inhibited. Overall, these results provide a new method to create hybrid DNA/RNA architectures for biosensing, while safeguarding the integrity of RNA sequences.

To assess the stability of RNA under native and denaturing conditions, we first used commercially available RNA from bacteriophage MS2 (3569 nt). Complementary oligos were designed with molecular barcodes as previously reported^6,7^ to create an identifiable current signature (termed “RNA ID”) for nanopore measurements. Each peak or “bit” in the RNA ID barcode is produced using six consecutive DNA dumbbells (Figure S1). The barcode “101” is specific to the MS2 sequence (Figure 1A).

**Figure 1:**
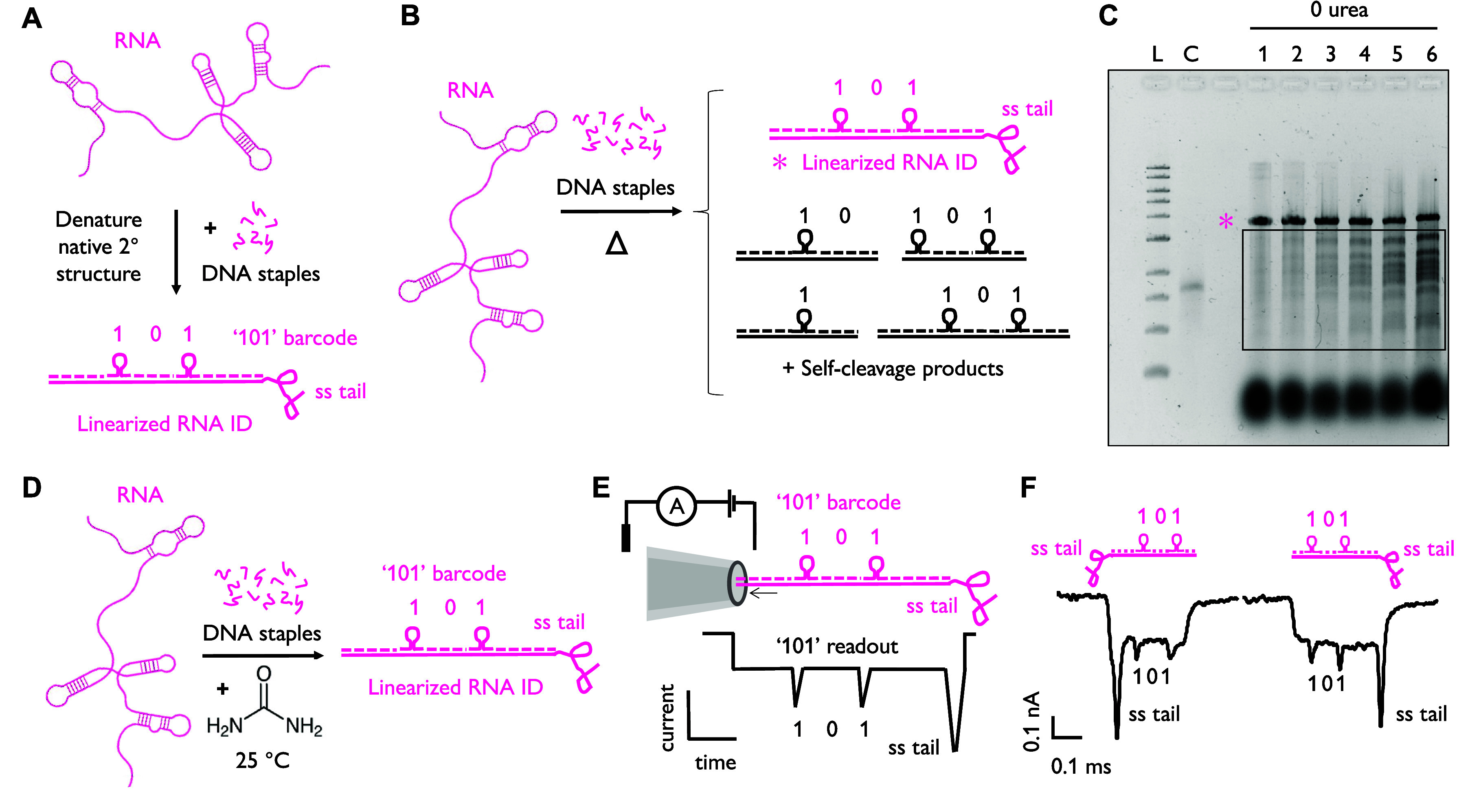
Hybridization of DNA to RNA to create RNA IDs for nanopore readout. (A) The denaturation of native RNA secondary structure allows complementary DNA staples to hybridize, forming the “101” MS2 RNA ID. (B) Using previous annealing protocols (*T* ≥ 70 °C in buffer/salt) to break up the RNA secondary structure results in RNA self-cleavage and thus truncated barcodes. (C) Agarose gel showing hybridization of complementary staples to MS2 at 70 °C in the presence of 100 mM LiCl, 10 mM tris at pH 7.5. While the RNA ID is formed (marked with a pink asterisk), the self-cleavage products are evident (black box), hampering RNA quantification. Lanes 1–6: 15 min, 1, 2, 4, 8, and 12 h incubation at 70 °C, respectively, Lane C (control): unduplexed MS2. (D) Scheme depicting urea-facilitated hybridization of DNA to MS2 RNA, forming the “101” ID at 25 °C. (E) Predicted nanopore signal of “101” RNA ID. (F) Real nanopore events showing the correct 101 ID. The RNA ID may enter the nanopore in either direction, leading to the current signatures depicted.

To create the RNA ID, the RNA strand is combined with a 5-fold excess of DNA complements to ensure complete hybridization (Figure 1D). The pH is held at 7.5 using 10 mM tris buffer. When MS2 RNA and its complements are heated to 70 °C to break up the RNA’s native secondary structure, self-cleavage occurs (Figure 1 B,C). This self-cleavage happens even in the absence of divalent cations, which would catalyze the reaction.^16,17^ Upon thermal annealing, the degradation products of RNA self-cleavage are apparent, appearing in the AGE as discrete higher mobility bands and smearing (Figure 1C). Even at the shortest sampled annealing time (15 min), degradation is evident. As such, thermal denaturation fails to produce the correct structure without RNA degradation.

To denature the RNA secondary structure without heating, we employed 5 M urea in the assembly solution. Urea-induced denaturation of RNA secondary and tertiary structure is dependent on salt concentration.^18^ As previously mentioned, divalent cations such as Mg^2+^ are not suitable here due to their catalytic action in the self-cleavage of RNA. Instead, we opted for monovalent cations and examined MS2 RNA integrity in varying concentrations of Li^+^, Na^+^, and K^+^. AGE results show no difference between the cations in terms of RNA degradation (Figure S2). We selected Li^+^, which has been shown to most effectively slow the translocation of DNA/RNA in nanopore experiments.^19^ Increasing the time between peaks facilitates the error-free readout of the RNA IDs. For all experiments presented herein, the concentration of Li^+^ during hybridization is held constant at 100 mM. Under these conditions, incubating the RNA/DNA at 25 °C in the presence of 5 M urea results in the formation of MS2 RNA IDs, as evidenced by the single-molecule nanopore traces, which clearly depict the “101” barcode (Figure 1 F).

Using 5 M urea in the assembly mixture allows hybridization at 25 °C. As a result of this lower temperature, no detectable, discrete self-cleavage products by AGE, even after 12 h of incubation (Figure 2A). A decrease in the intensity of the DNA staple band relative to the “no urea” control confirms the successful hybridization of DNA complements to the target RNA in these conditions, demonstrating that the multivalent DNA/RNA interactions overcome the denaturing effect of the urea. Without the inclusion of urea, incubation at 25 °C does not produce a well-defined band by AGE. We thus conclude that the secondary structure of the native RNA cannot be displaced by the DNA complements without the denaturing effect of urea (Figure 2A).

**Figure 2:**
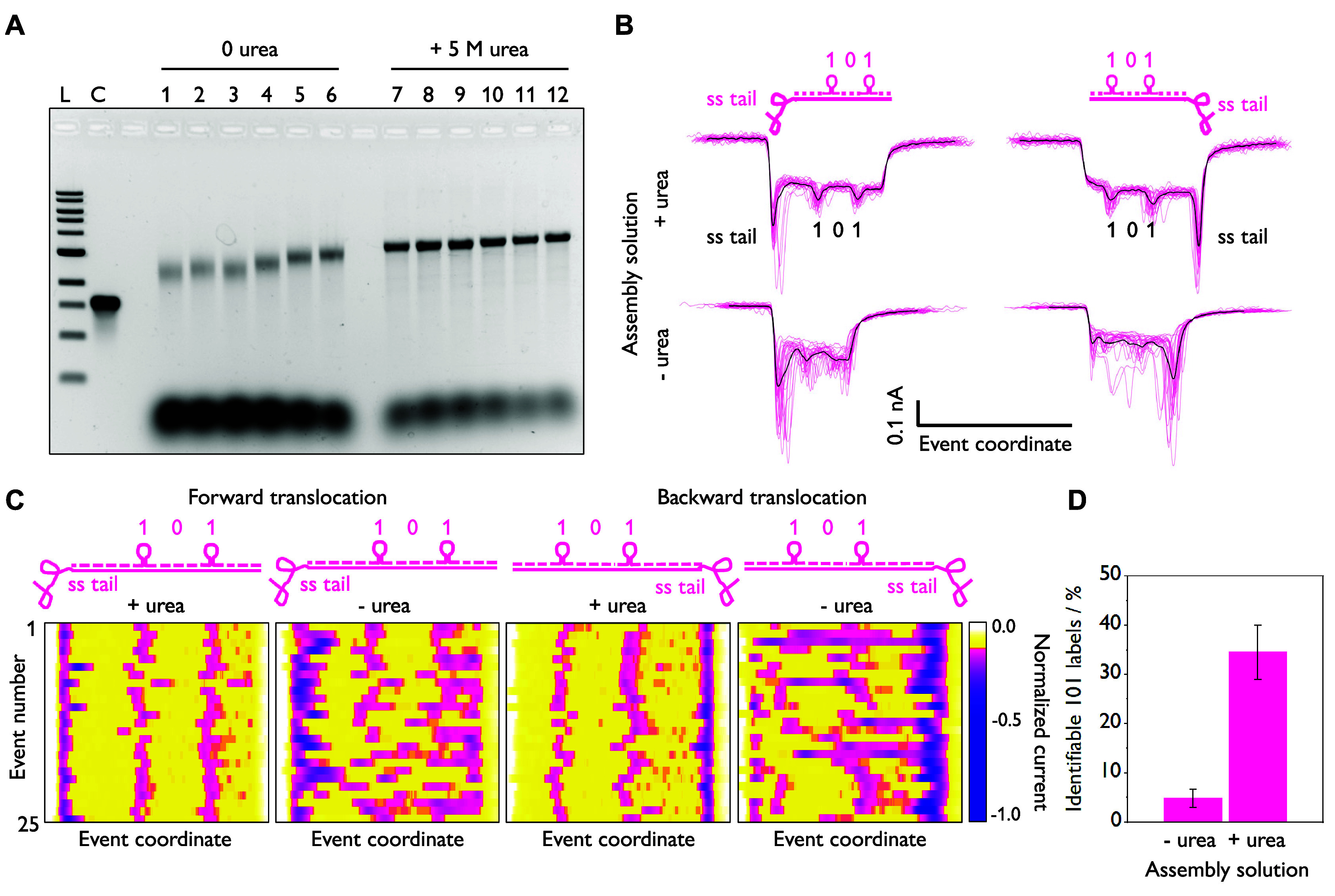
Characterization of urea-enabled hybridization of DNA to RNA. (A) Agarose gel showing hybridization of complementary staples at room temperature with and without 5 M urea. Lanes 1–6: 15 min, 1, 2, 4, 8, and 12 h incubation, respectively, with no urea. Lanes 7–12: 15 min, 1, 2, 4, 8, and 12 h incubation, respectively, in the presence of 5 M urea. Lane C (control): unduplexed MS2. (B) Overlayed nanopore signals (pink) for the first 25 unfolded barcoded events in each translocation direction with (top) and without (bottom) 5 M urea in the assembly solution. Black trace signifies the mean of the 25 events for each plot. (C) Two-dimensional plot representing the first 25 unfolded, barcoded events in the “101” sample prepared with and without 5 M urea in the assembly solution, with the color indicating the current value. Events are aligned to the initial current drop, which indicates entry of the analyte into the nanopore. (D) Populations of correctly identified 101 barcodes from samples assembled without and with 5 M urea. Three separate nanopore experiments were performed for each sample. Error bars indicate the standard error; the center is the mean. *N* ≥ 2000 events for each replicate.

Next, nanopore sensing was used to quantify the relative folding efficiencies of RNA IDs under different conditions (Figure 2B–D). In Figure 2B, the first 25 unfolded “101” single-molecule translocation events for assembly at 25 °C for 12 h with and without urea are overlaid. When urea is employed, the correct 101 current signatures are remarkably uniform (Figure 2B) and the mean event clearly displays the designed barcode. The 300 nt single-stranded tail also produces an additional, deep current blockade, commensurate with previous reports.^20^ By comparison, without urea in the assembly solution, the resulting nanopore traces are variable, and the mean event does not reproduce the designed readout. Comparing these data sets shows that denaturing conditions result in more homogeneous RNA IDs due to the minimization of both native RNA secondary structure and self-cleavage activity.

The homogeneity of nanopore events afforded by our urea protocol is further illustrated in Figure 2C. The first 25 unfolded “101” events in each translocation direction for assembly at 25 °C for 12 h with and without 5 M urea are compared. The current is encoded in the color scale, and the length and plateau depth of each event is normalized (see the Supporting Information for details). By stacking individual events side-by-side, it is clear that the chemical annealing with urea yields consistent nanopore events with clear labeling and aligned barcode peaks. In contrast, without urea in the assembly solution, barcodes are heterogeneous and challenging to identify. Moreover, the urea-enabled annealing returns the correct 101 current signature in ∼35% of the total events, whereas only 5% of events are identified as having the 101 barcode in the absence of urea (Figure 2D). Thus, in addition to providing more homogeneous events, the use of urea increases the proportion of identifiable current signatures 7-fold. Notably, the products are stable in urea even over several days when stored at 4 °C (Figure S6).

Having established that chemical annealing with urea results in restructured RNA IDs for nanopore detection, we expanded this methodology to use in multiplexed RNA sensing. To this end, we used total RNA from human epithelial cells and designed DNA barcodes specific to the ribosomal 18S (1869 nt, RNA ID “111”) and 28S (5070 nt, RNA ID “11111”). The native structures of these rRNA sequences and the RNA ID designs are depicted in Figure 3A and B.^21^

**Figure 3:**
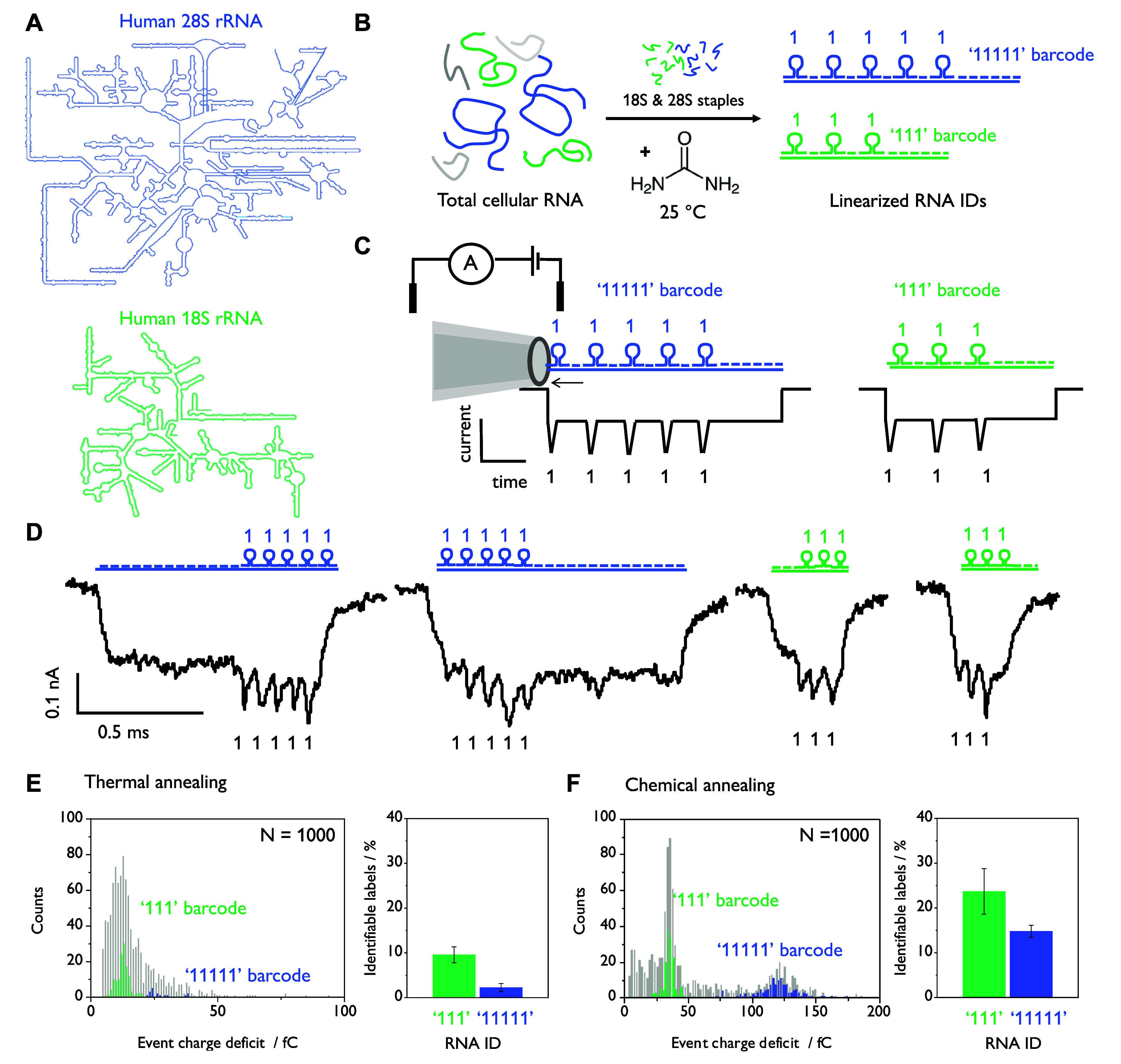
Multiplexed nanopore detection of rRNA. (A) Secondary structures of human 18S and 28S rRNA. (B) Scheme illustrating urea-facilitated hybridization of specific DNA barcodes to 18S and 28S within total cellular RNA, resulting in RNA IDs. (C) Predicted nanopore events for the 28S (“11111”) and 18S (“111”) RNA IDs. (D) Real nanopore events showing the “11111” and “111” IDs, corresponding to the 28S and 18S, respectively. (E) Thermal annealing produces the 18S “111” ID in fair yield but degrades the 28S, which is identified in only 2% of single-molecule events. (F) When chemically annealed with 5 M urea, significantly more of both the 18S and 28S rRNA are identifiable, with less RNA degradation. Three separate nanopore experiments were performed for each sample. Error bars indicate the standard error. The center is the mean. *N* = 1000 events for each replicate.

Total RNA was incubated with DNA barcode sequences targeting both the 18S and 28S rRNA. The same protocol as described for MS2 (25 °C, 5 M urea, pH 7.5, 10 mM tris, 100 mM LiCl, 12 h, 5× excess of DNA staples) was used. AGE confirmed the hybridization of the DNA complements and RNA stability over time (Figure S5). Nanopore sensing enabled the clear identification of both the “111” and “11111” RNA IDs from within the complex mixture of RNAs (Figure 3C, D), demonstrating that our chemical denaturation method can be used to restructure complex RNA motifs from human samples.

For the chemically annealed 28S RNA ID, while the “11111” barcode was readily identified using nanopore sensing, an additional peak appeared in >95% of single-molecule events upon hybridization for 12 h at room temperature (Figure S9). Based on the distance from the barcode, we attribute this signal to the GC-rich and highly self-complementary tract from nt ∼3000–3500, which is ostensibly too stable to be reshaped using our original methodology. This peak was also visible when a standard, thermal annealing protocol was used (heat to 70 °C for 30 s then slowly cool to 4 °C 45 min without urea^7^), further illustrating the stability of the underlying RNA secondary structure (Figure S10). We found that we could diminish this peak through the addition of a short heating step (3 min at 80 °C) prior to 12 h at 25 °C in 5 M urea (Figure S11). This result highlights how short thermal denaturation and prolonged chemical annealing may be used in tandem to reshape RNA molecules with highly stable native secondary structures while minimizing RNA degradation.

We next benchmarked our optimized chemical annealing protocol for the rRNA (3 min at 80 °C, rapid cooling to 25 °C, then 12 h incubation, all in 5 M urea) by comparing it against the previous thermal annealing method to generate RNA/DNA hybrids (70 °C with slow cooling to 4 °C over 45 min pH 7.5, 10 mM tris, 100 mM LiCl, 5x excess of DNA staples, no urea).^7^ The first 1000 nanopore events for each of the thermal and chemical annealing methods are compared in Figure 3. While “111” and “11111” RNA IDs corresponding to the 18S and 28S rRNA are still identifiable when thermal annealing is employed, the population shifts toward shorter events with lower mean currents, suggesting RNA degradation. Particularly, the “11111” RNA ID denoting the 28S rRNA, which has multiple reported self-cleavage sites,^22^ was only observed in <5% of events across three replicates in different nanopores.

Using the chemical annealing method, 24% of events corresponded to the “111” barcode and 15% of events matched the “11111” (means of three replicates in different nanopores). This data represents a doubling of the number of correctly identified events for the 18S and 6.5-fold improvement in the number of “11111” barcodes, underlining how native RNA secondary structure can impact the correct folding of hybrid barcodes if not fully denatured *a priori*. The stark difference in the quantity of identified “11111” events between the thermally and chemically annealed samples also emphasizes how RNA degradation may bias the relative quantification of biomarkers if assembly procedures are not optimized.

In summary, we have demonstrated a chemical annealing protocol that promotes the hybridization of DNA staples to RNA by first denaturing RNA’s native secondary structure. Employing urea, this procedure enables the controlled restructuring of RNA molecules for biosensing applications. Unlike previous reports wherein DNA origami was folded in the presence of chemical denaturants,^23^ our method does not require a decreasing concentration of urea over time to generate folded structures. Instead, capitalizing on the cooperative binding of DNA complements, we formed the hybrid RNA IDs directly in 5 M urea. This protocol is compatible with single-molecule nanopore sensing, facilitating the direct identification and quantification of RNA targets in complex samples without enzymatic amplification. Because heating equipment is not required (depending on the initial secondary structure), chemical annealing may be suitable for field testing of RNA samples. We envisage that this method will support the development of new biosensors based on DNA/RNA hybridization and will further the integration of RNA into the field of DNA nanotechnology.

## Supporting Information Available

Additional information including AGE data, nanopore data, oligonucleotide sequences, materials, and methods (PDF)
