# Supplementary material for: Chemical Annealing Restructures RNA for Nanopore Detection
Source: J Am Chem Soc. 2024 May 1;146(19):12919–24. doi: 10.1021/jacs.4c03753 (PMC11099964; doi:10.1021/jacs.4c03753)
Supplement: Supplementary file 1 — ja4c03753_si_001.pdf [file ja4c03753_si_001.pdf]

Supporting Information

Chemical annealing restructures RNA for nanopore detection

Casey M. Platnich, Max K. Earle, Ulrich F. Keyser

Cavendish Laboratory, University of Cambridge, CB3 0HE Cambridge, United Kingdom

Contents:

1. Materials
2. RNA ID design
3. Assembly of hybrids
4. Native agarose gel electrophoresis
5. Nanopore fabrication
6. Nanopore measurements
7. Nanopore data analysis
8. Supplementary figures
9. Oligonucleotide sequences
10. References

1. Materials.

Glass capillaries (inner diameter 0.2 mm, outer diameter 0.5 mm) were purchased from Sutter Instruments (California, USA). DNA oligonucleotides were purchased from Integrated DNA Technologies as OligoPools (unmodified, 50 pmol scale). The oligo sequences are listed in Tables S1-S3. MS2 RNA (3569 nt in length) was purchased from Roche and used without further purification. PDMS used in the fabrication of nanopore chips is Sylgard 184 silicone elastomer kit (The Dow Chemical Company, MI, USA). All water is DEPC-treated nuclease-free water (Merck). Stock 20x TE buffer (nuclease-free) was purchased from Fisher Scientific. All buffers were filtered with 0.22 μm Millipore syringe filter units (Merck).

2. RNA ID design

Each bit in the barcode is composed of six consecutive DNA dumbbells, which has been previously shown to provide good signal to noise for DNA/RNA hybrids.[1] Each dumbbell strand is 48 nts in length, with 28 nt making up the dumbbell unit and 10 nt complementary to the RNA on either side of the dumbbell. This total binding footprint of 20 nt on the RNA ensures the stability of the product at room temperature. The 28 nt sequence which forms the dumbbell is kept constant for all RNA IDs (5′-TCCTCTTTTGAGGAACAAGTTTTCTTGT-3′, full sequences for all strands are provided in Section S9). In total, each bit has a footprint of 120 nt.

Barcodes are placed towards one end of the RNA sequence, as this asymmetry allows the direction of translocation to be readily identified. Additionally, barcodes are spaced at least 278 nt (~ 72 nm, assuming a A-form helix) from the end of the RNA strand, to enable differentiation between bit peaks and RNA folding. Separation of at least 278 nm between bits ensures that the peaks can be temporally resolved within the nanopore.

The ability to form uniform barcodes in high yield is dependent on the initial secondary structure of the RNA (Figures S9-S11): when designing RNA IDs, care should be taken to avoid placing barcodes in RNA tracts with significant local secondary structure, when possible.

3. Assembly of hybrids.

Assembly was performed at 20 nM of the RNA strand with DNA complements added at a 5x excess (100 nM). 1 x TE (pH 7.5) was used to buffer and LiCl (100 mM final) was used to screen the negative charges of the phosphate backbone. Urea was added to a final concentration of 5 M. The sample was then mixed by flicking, microcentrifuged, and placed in a thermocycler (Applied Biosystems™ ProFlex™ PCR System, 3 x 32-well) to incubate at a given temperature for a controlled time period, after which the temperature was held at 4 °C until ready to filter. The sample was filtered (100 kDa Amicon filter, Merck) and its concentration was measured in a Thermo Scientific Nanodrop™ 2000 Spectrophotometer.

4. Native agarose gel electrophoresis.

Agarose gel electrophoresis (0.8% w/v agarose) was conducted using a BioRad Sub-Cell GT electrophoresis cell with 1 x TBE (with 0.05% sodium hypochlorite solution) as the running buffer. Gels were run for 2.5 hours at 70 V on ice. Samples are prepared to 150 ng RNA in 10 uL for loading and consist of the sample, 1 x TBE buffer and 1 x purple loading dye (no SDS). The ladder used is the 1 kB ladder from New England Biolabs. After running, gels were stained using GelRed (Biotium) and imaged using the GelDoc-It™ (UVP). Gel images were processed using Fiji (ImageJ) by inverting the grayscale and subtracting the homogenous background with 100-150 pixels rolling ball.

5. Nanopore fabrication.

Quartz glass capillaries were pulled to the desired diameter (~10 nm) using a laser-heated pipette puller (P-2000, Sutter Instrument, California, USA). The parameters used were: HEAT=475, FIL=0, VEL=25, DEL=170, PUL=225. Please refer to the P-2000 manual for the explanation of the parameters. After pulling, nanopore were cut to length and positioned within a custom polydimethylsiloxane (PDMS) chip with 8 pores per chip, which was then plasma bonded (Femto, Diener Electronic, Germany) to a glass slide. PDMS was used to seal with pores within the chip and baked at 120 °C for 2 hours for curing. After baking, chips are placed in the plasma cleaner for 5 minutes to ensure a hydrophilic surface layer. 1 x TE buffer with 4 M LiCl with a pH of 9.4 (adjusted with LiOH) was then added to the central reservoir as well as the outer chambers.

Samples for nanopore measurements were diluted to ~ 300 pM in 1 x TE, 4 M Li (pH 9.4) and injected into the central reservoir.

6. Nanopore measurements.

An Axopatch 200B (Molecular Devices, CA, USA) was used to perform nanopore measurements. The signal was filtered with an external Bessel filter (Frequency Devices) at 50 kHz and digitized at a 250 kHz sampling rate with a data card (PCI-6251, National Instruments). Two Ag/AgCl electrodes were prepared by curing 1-mm Ag wires in a 10% solution of NaClO. These were then inserted into the central reservoir (cis) and the outer chamber (trans) to create an electrical circuit across the nanopore. Current-voltage curves were measured from -600 mV to 600 mV to estimate nanopore size prior to measurements. Approximate diameters were calculated from their conductance as previously described. Pores with a maximum current of ~ 10 nA and a root-mean-square (RMS) noise below 7.5 pA were selected for measurements.

7. Nanopore data analysis.

The data was analysed using an in-house peak finding algorithm in LabVIEW. Individual events were isolated by thresholding by duration, current drop, and event charge deficit

(ECD). From these isolated events, we can then discriminate between folded and unfolded events, as well as determining the barcode by identifying the downward peaks within an event.

To generate the overlaid and mean event traces in Figure 2B, a custom Python script was employed. In this analysis, every translocation event in the relevant dataset (25 events per set) was subjected to a simple scaling in the time direction such that the point at which each current drops below -0.1 nA is aligned on the x-axis. We assume that translocation speeds of identical carriers in a nanopore experiment may vary but that this variability is adequately described by a constant scaling transformation. The data of each scaled event were then interpolated at equally spaced points, with a total number of points equal to the number of points in the longest event trace. This ensured that each event was oversampled and therefore no signal information was lost under this scaling transformation. The scaled and interpolated events were then plotted and directly overlaid with one another. The mean event was calculated by averaging the current values of every event for each point on the event coordinate axis.

To generate the two-dimensional event number-event coordinate-current plots in Figure 2C, each event in the four datasets (25 events per set, the same events as in Figure 2B) was stretched in the time direction (as for the plots in Figure 2B). The events were then further scaled on the current axis such that their first level plateaus (i.e. the current corresponding to the DNA/RNA duplex) were equal (value of -0.1104, normalized current). This normalization scheme was selected such that the deepest current peak from any of the four datasets reached a scaled value of exactly -1.0, allowing every plot to use the same colourmap. The colourmap was designed so that each dataset would have its deepest point within the blue level. Any current value between the maximum positive current and 0 is white, the event plateau (between -0.1104 and -0.1322, normalized current) is yellow, and any value in between the plateau and the first blue level is pink. The x-axes of these plots begin 20 data points before the start of the event plateau and end 20 data points after the event plateau has ended.

8. Supplementary figures

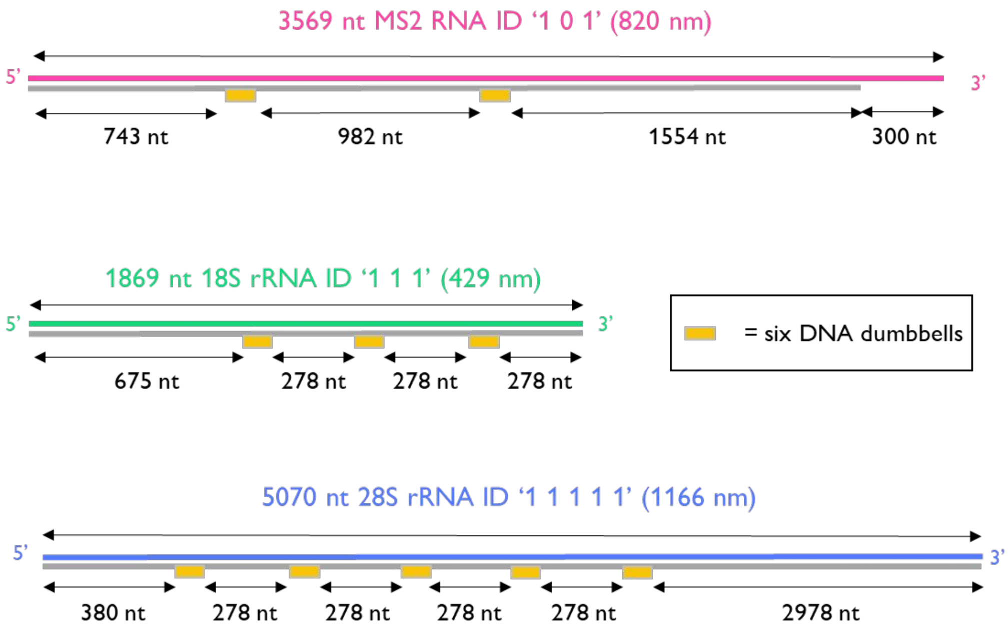

Figure S1. Designs for RNA IDs. Approximate lengths for the constructs are given assuming an A-form helical geometry.

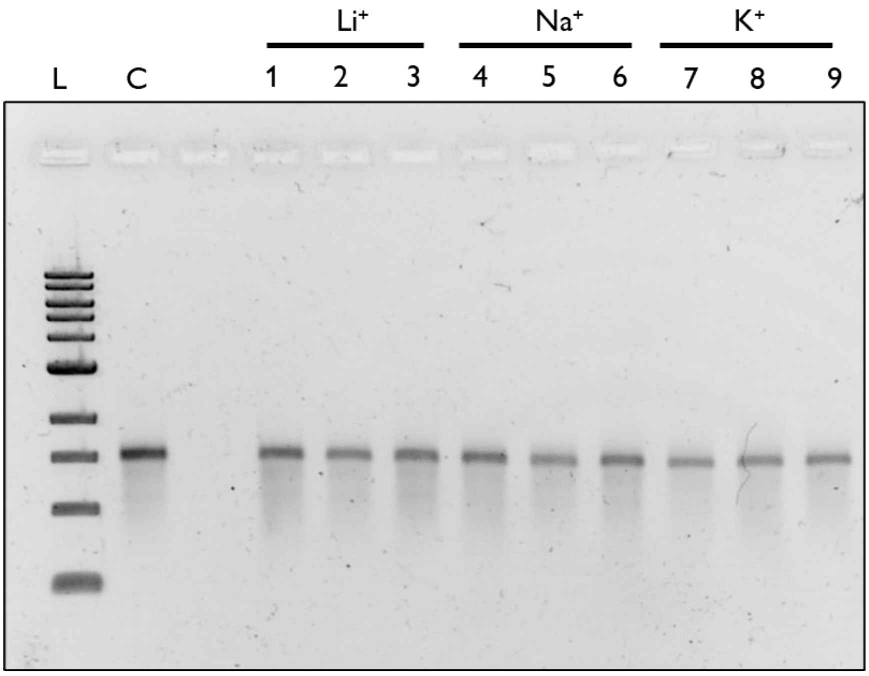

Figure S2. Electrophoretic analysis of MS2 RNA incubated at 70 C for 15 minutes then cooled to 4 C in a single step with different monovalent salts. Lane 1: LiCl, 100 mM. Lane 2: LiCl, 250 mM. Lane 3: LiCl 500 mM. Lane 4: NaCl, 100 mM. Lane 5: NaCl, 250 mM. Lane 6: NaCl, 500 mM. Lane 7: KCl, 100 mM. Lane 8: KCl, 250 mM. Lane 9: KCl, 500 mM. L: 1 kB DNA ladder (New England Biolabs). C: control, MS2 RNA directly from freezer storage.

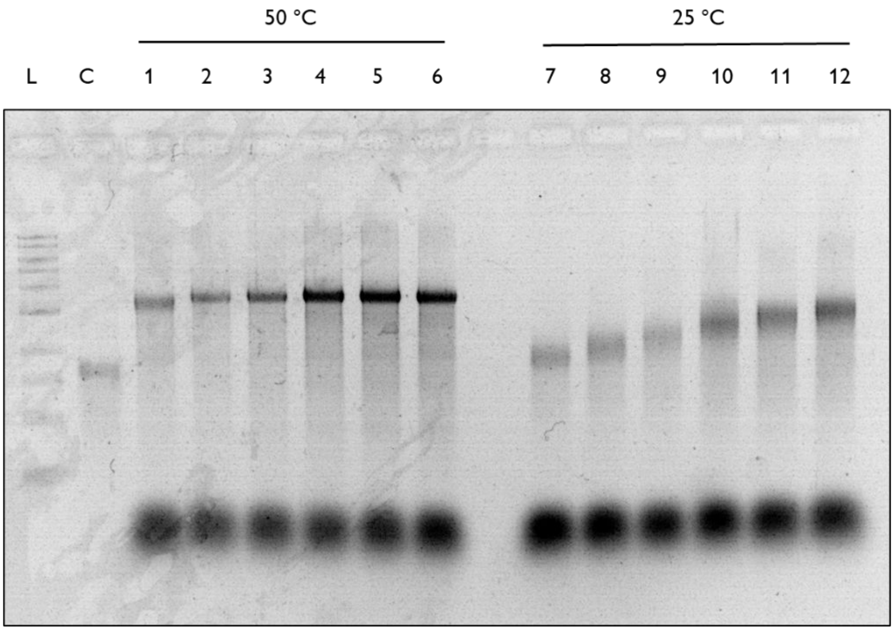

Figure S3. Electrophoretic analysis of DNA-duplexed MS2 RNA hybridized in the absence of urea at 50 °C (left) and 25 °C (right). Lanes: L = 1 kB DNA ladder (New England Biolabs). C = control, MS2 RNA directly from freezer storage. 1-6 = incubation at 50 °C for 15 minutes, 1 hour, 2 hours, 4 hours, 8 hours, and 12 hours (respectively). 7-12 = incubation at 25 °C for 15 minutes, 1 hour, 2 hours, 4 hours, 8 hours, and 12 hours (respectively). An excess of DNA staples can be observed at the bottom of the gel, which was run prior to Amicon filtration.

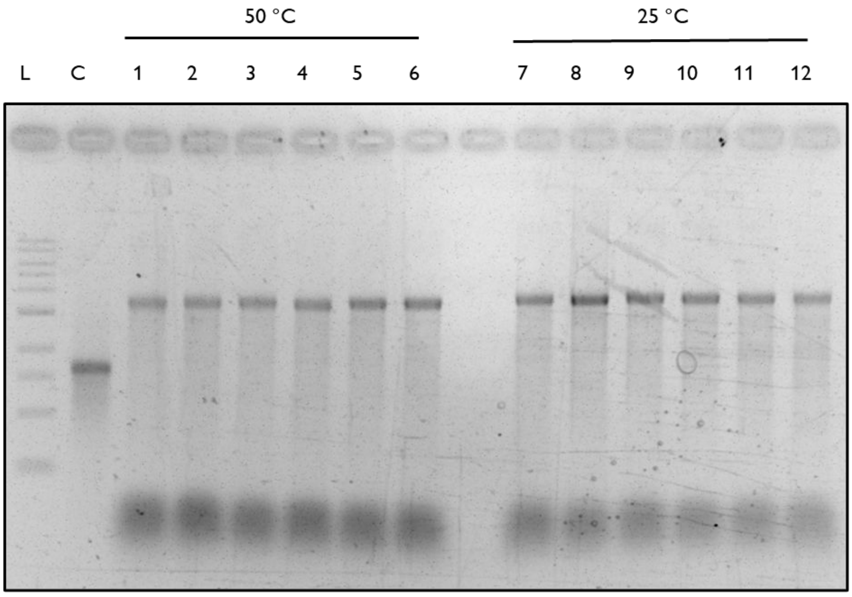

Figure S4. Electrophoretic analysis of DNA-duplexed MS2 RNA hybridized in 5 M urea at 50 °C (left) and 25 °C (right). Lanes: L = 1 kB DNA ladder (New England Biolabs). C = control, MS2 RNA directly from freezer storage. 1-6 = incubation at 50 °C for 15 minutes, 1 hour, 2 hours, 4 hours, 8 hours, and 12 hours (respectively). 7-12 = incubation at 25 °C for 15 minutes, 1 hour, 2 hours, 4 hours, 8 hours, and 12 hours (respectively). An excess of DNA staples can be observed at the bottom of the gel, which was run prior to Amicon filtration.

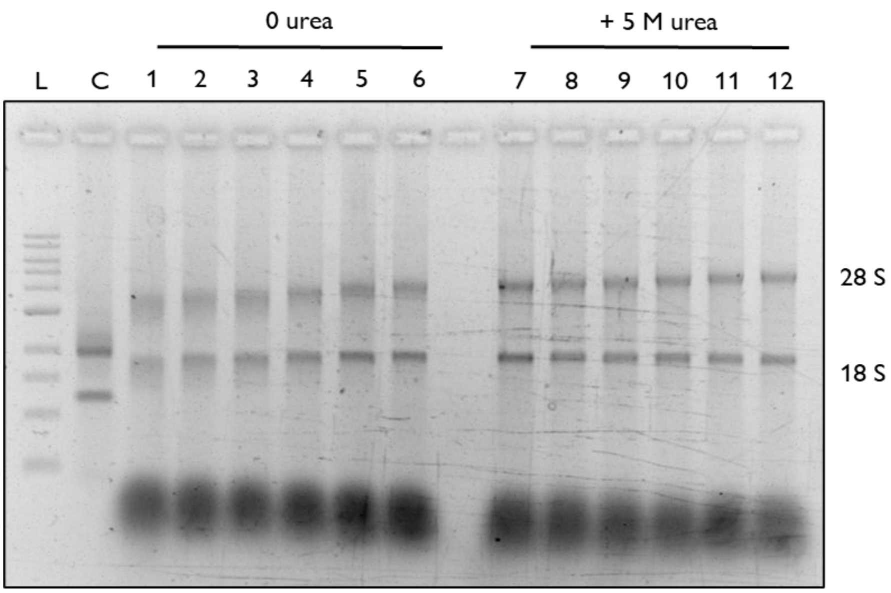

Figure S5. Electrophoretic analysis of DNA-duplexed 18S and 28S ribosomal RNA hybridized in the absence (left) and presence (right) of 5 M urea at 25 °C. Lanes: L = 1 kB DNA ladder (New England Biolabs). C = control, total cellular RNA directly from freezer storage. The two prominent bands correspond to the 28S (upper) and 18S (lower). 1-6 = 25 °C incubation for 15 minutes, 1 hour, 2 hours, 4 hours, 8 hours, and 12 hours (respectively), without urea. 7-12 = 25 °C incubation for 15 minutes, 1 hour, 2 hours, 4 hours, 8 hours, and 12 hours (respectively), with 5 M urea. Bands corresponding to the duplexed 18S and 28S are labelled. An excess of DNA staples can be observed at the bottom of the gel, which was run prior to Amicon filtration.

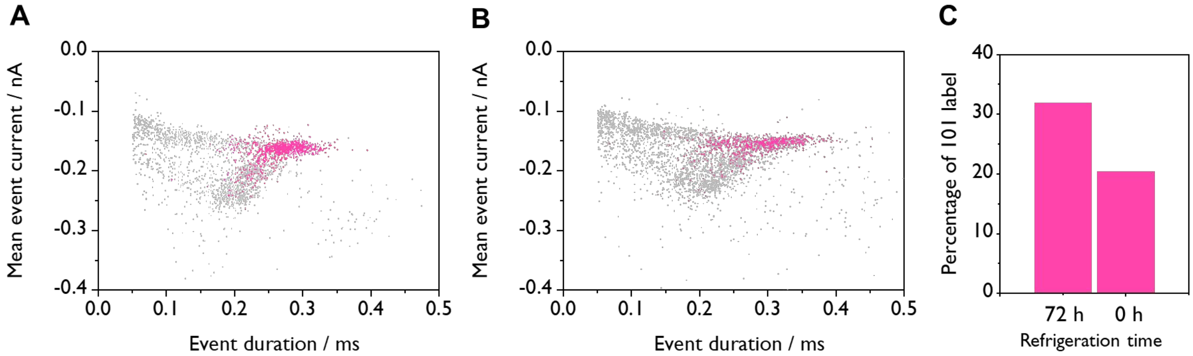

Figure S6. Nanopore data for MS2 RNA IDs chemically annealed in 5 M for 4 h at 25°C. (A) Sample stored for 72 h at 4 °C in 5 M urea after the initial assembly. (B) Sample measured directly after 4 h at 25°C. (C) Comparison of number of identifiable labels before and after 72 h of refrigeration. The results show that the assembly improves over time incubating at 4°C in urea.

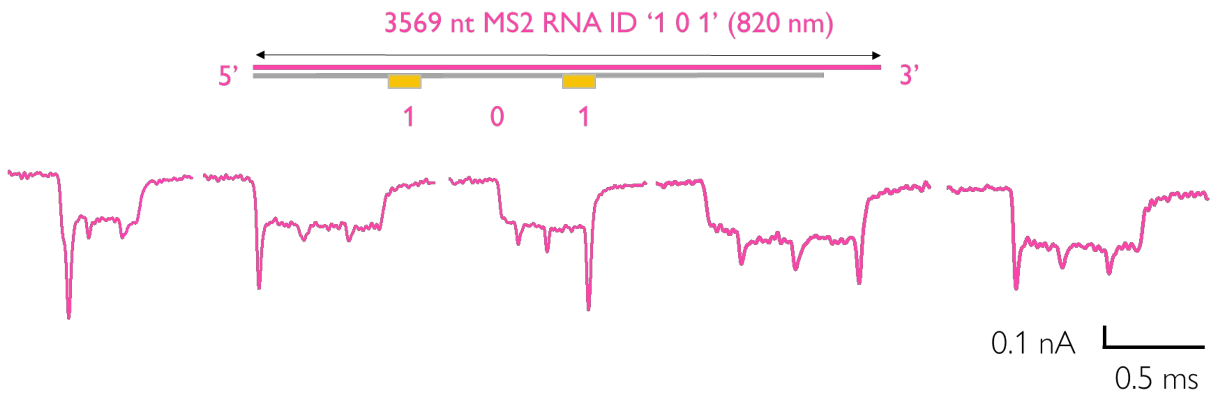

Figure S7. Example nanopore translocation events for MS2 RNA ID "1 0 1". Samples were incubated at 25 °C for 12 hours with 5 M urea.

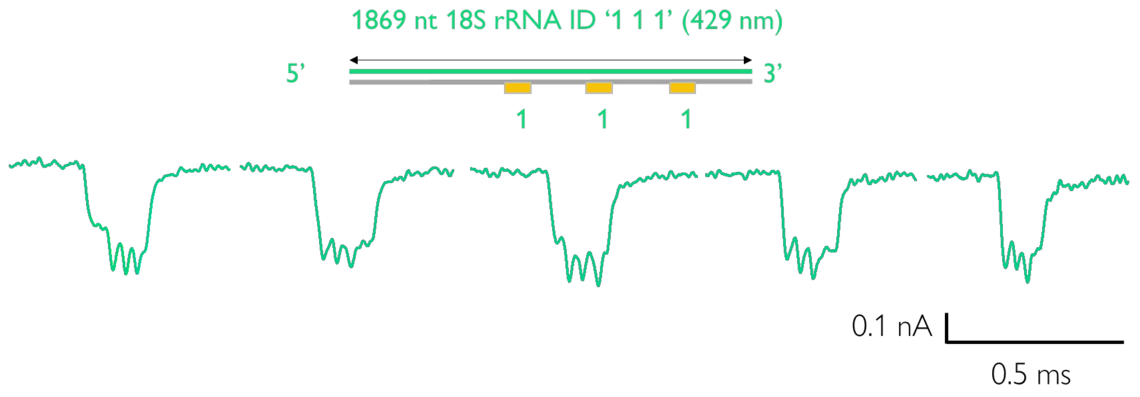

Figure S8. Example nanopore translocation events for 18S rRNA ID "1 1 1". Samples were incubated at 25 °C for 12 hours with 5 M urea.

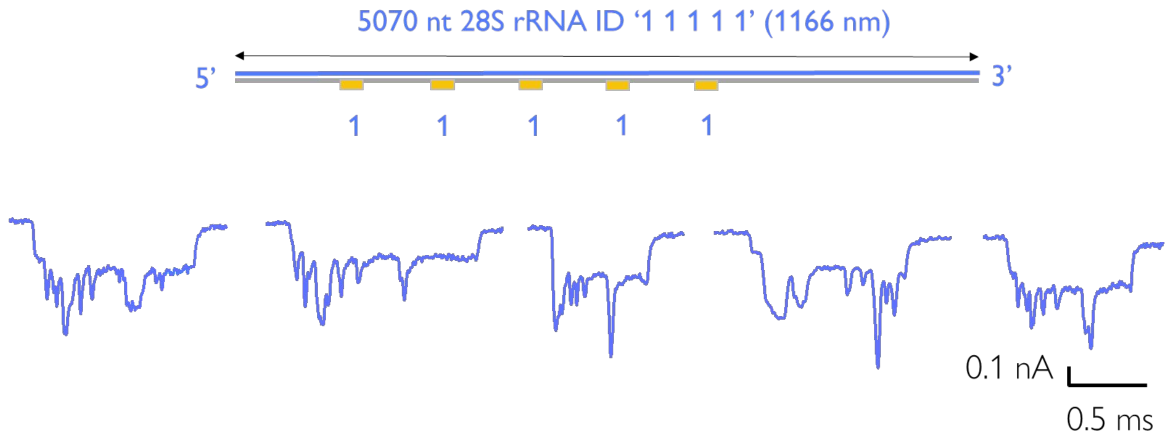

Figure S9. Example nanopore translocation events for 28S rRNA ID "1 1 1 1 1". Samples were incubated at 25 °C for 12 hours with 5 M urea. An extra, broad peak is visible in a similar position in all traces, suggesting an area of local RNA secondary structure.

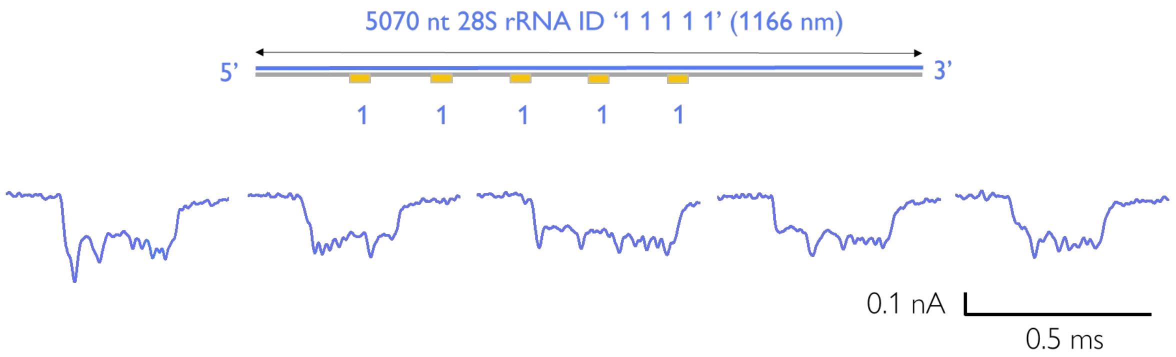

Figure S10. Example nanopore translocation events for 28S rRNA ID "1 1 1 1 1". Samples were incubated at 70°C for 30 s and then gradually cooled down (-0.5 °C/cycle, 90 cycles each 30 s) over 45 minutes to room temperature, then dropped to 4 °C. An extra, broad peak is visible in a similar position in all traces, suggesting an area of local RNA secondary structure.

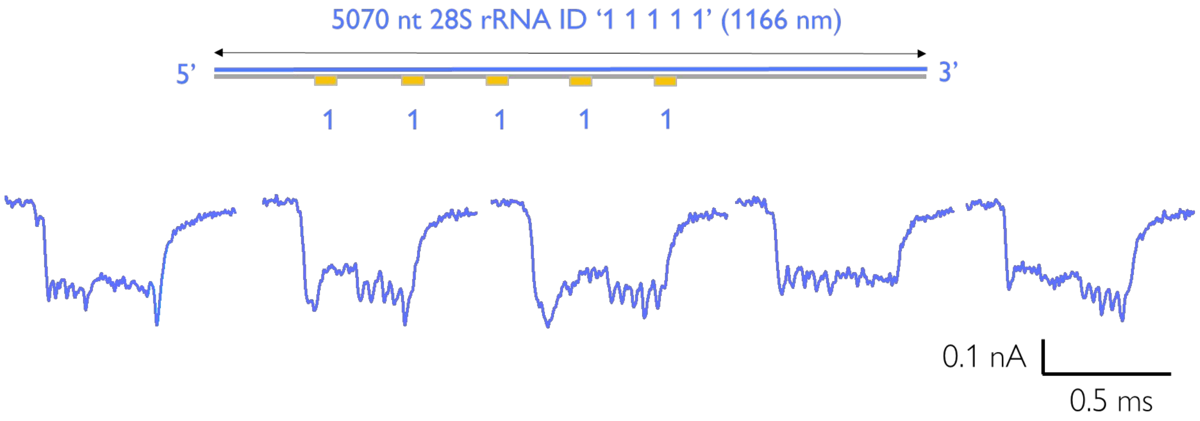

Figure S11. Example nanopore translocation events for 28S rRNA ID "1 1 1 1 1". Samples were heated to 80°C for 3 minutes then incubated at 25 °C for 12 hours with 5 M urea.

9. Oligonucleotide sequences.

Table S1. Oligonucleotide sequences for DNA strands complementary to MS2 RNA and producing the 101 RNA ID. Dumbbell-containing sequences are highlighted in red.

NAME	SEQUENCE (5' TO 3')	LENGTH (NT)
MS2_101_1	GGCATTAGCTCGACAGGAAGTTGAGCAGGACCCCGAAAGGGGTCCCACCC	50
MS2_101_2	GCTACCTACAGCGATAGCCATGGTAGCGTCTCGCTAAAGACATTAAAAAT	50
MS2_101_3	AAGGGTACTAAAAGCTCGCACAGGTCAAACCTCCTAGGAATGGAATTCCG	50
MS2_101_4	CCGTCCGCGTAAACGCGAACGGAGGGGACGAAGGTCTCGTTCTCCCTATC	50
MS2_101_5	GAGTCCAGTTCGAACGATATTTTAAAGAGAATGAGTTATCTTCAGTCTCA	50
MS2_101_6	GGGGTAGTGCCACTGTTTCGTTTTGGCCCCAGTCGAGTTAAAACGACCGG	50
MS2_101_7	GCACCTTGATCTATCGATGTGACACTTAACGCCCCCCGTGAATACGGAGA	50
MS2_101_8	CCGGCTTTCTCCTCGTACGGGCGACCCCACGATGACCCACTTCGCTTGTAG	51
MS2_101_9	CACTCCGTTCCCTACAACGAGCCTAAATTCATATGACT	38
MS2_101_10	CGTTATAGCGGACCGCGTGTCTGATCCACGGCGCACAT	38
MS2_101_11	TGGTCTCGGACCAATAGAGCCGCTCTCAGAGCGCGGGG	38
MS2_101_12	GGTAACGGTTGCTTGTTCAGCGAACTTCTTGTAAGGCG	38

MS2_101_13	CTGCATCCTGCAACTTGTGCCCCATAGGAGCACCGTTG	38
MS2_101_14	GAGAACGTGCATTGCCCAAACAACGACGATCGGTAGCC	38
MS2_101_15	AGAGAGGAGGTTGCCAATAAGGCTACGGATGCTGGTTT	38
MS2_101_16	GTAAAACATCCGGATCCCATGACAAGGATTTGTCATGT	38
MS2_101_17	AAGAAACCTTCTCTATTTATCTGACCGCGATCACCATT	38
MS2_101_18	CGCCTCCCGTTCCTCTTTTGAGGAACAAGTTTTCTTGTAGCTTAGCGA	48
MS2_101_19	TAGCTAAGGTTCCTCTTTTGAGGAACAAGTTTTCTTGTACGACGGGTC	48
MS2_101_20	GCCTCGTCATTCCTCTTTTGAGGAACAAGTTTTCTTGTTACCAGAACC	48
MS2_101_21	TAAGGTCGGATCCTCTTTTGAGGAACAAGTTTTCTTGTTGCTTTGTGA	48
MS2_101_22	GCAATTCGTCTCCTCTTTTGAGGAACAAGTTTTCTTGTCCTTAAGTAA	48
MS2_101_23	GCAATTGCTGTCCTCTTTTGAGGAACAAGTTTTCTTGTTAAAGTCGTC	48
MS2_101_24	ACTGTGCGGATCACCGCTTCCAGTAGCGACAG	32
MS2_101_25	AAGCAATTGATTGGTAAATTTCGAGAGAAAGATCGCGA	38
MS2_101_26	GGAAGATCAATACATAAAGAGTTGAACTTCTTTGTTGT	38
MS2_101_27	CTTCGACATGGGTAATCCTCATGTTTGAATGGCCGGCG	38
MS2_101_28	TCTATTAGTAGATGCCGGAGTTTGCTGCGATTGCTGAG	38
MS2_101_29	GGAATCGGGTTTCCATCTTTTAGGAGACCTTGCATTGC	38
MS2_101_30	CTTAACAATAAGCTCGCAGTCGGAATTCGTAGCGAAAA	38
MS2_101_31	TTGGAATGGTTAGTTCCATATTTAAGTACGAACGCCAT	38
MS2_101_32	GCGGCTACAGGAAGCTCTACACCACCAACAGTCTGGGT	38
MS2_101_33	TGCCACTTTAGGCACCTCGACTTTGATGGTGTATTTGC	38
MS2_101_34	GATTCTGCGCAGAGCTCTGACGAACGCTACAGGTTACT	38
MS2_101_35	TTGTAAGCCTGTGAACGCGAGTTAGAGCTGATCCATTC	38
MS2_101_36	AGCGACCCCGTTAGCGAAGTTGCTTGGGGCGACAGTCA	38
MS2_101_37	CGTCGCCAGTTCCGCCATTG	20
MS2_101_38	TCGACGAGAACGAACTGAGT	20
MS2_101_39	AAAGTTAGAAGCCATGCTTC	20
MS2_101_40	AAACTCCGGTTGAGGGCTCT	20
MS2_101_41	ATCTAGAGAGCCGTTGCCTG	20
MS2_101_42	ATTAATGCTAACGCATCTAA	20
MS2_101_43	GGTATGGACCATCGAGAAAGGAGACTTTACGT	32
MS2_101_44	ACGCGCCAGTTGTTGGCCATACGGATTGTACCCCTCGA	38
MS2_101_45	TGCATGGCTGAGATTTGGGCCTTAGCAGTGCCCTGTCT	38
MS2_101_46	CTCCACAGTCCACCCGTAGGGAGCGTCAACGCTTATGA	38
MS2_101_47	TGGACTCACCCGTTATTACGTCAGTAACTGTTCCTGAC	38
MS2_101_48	ATGTAGGAGCATCCCACGGGGGCCGTAAGGCCCTCGAG	38
MS2_101_49	CATGTTACCTACAGGTAGGAGCCAGTCGACAACGAATG	38
MS2_101_50	AGAAAGGCACCTTTTCCCACACTATACCTAGTGGGTTC	38
MS2_101_51	AAGATACCTAGAGACGACAACCATGCCAAACGTGCATC	38
MS2_101_52	GTTTATGTAAAACCATATCACGATACGTCGCGATATGT	38
MS2_101_53	TGCACGTTGTTCCTCTTTTGAGGAACAAGTTTTCTTGTCTGGAAGTTT	48
MS2_101_54	GCAGCTGGATTCCTCTTTTGAGGAACAAGTTTTCTTGTACGACAGACG	48
MS2_101_55	GCCATCTAACTCCTCTTTTGAGGAACAAGTTTTCTTGTTTGATGTTAG	48
MS2_101_56	TACCGACCTGTCCTCTTTTGAGGAACAAGTTTTCTTGTACGTACGGCT	48
MS2_101_57	CTCATAGGAATCCTCTTTTGAGGAACAAGTTTTCTTGTGAAACTCTTG	48
MS2_101_58	AAGGTGAACCTCCTCTTTTGAGGAACAAGTTTTCTTGTTTCGTAAGCA	48
MS2_101_59	TCTCATATGCACCCTGGATATCACTCATTAGT	32
MS2_101_60	GGTAACCAACCGAACTGCAACTCCAACCACCTGCCGGC	38
MS2_101_61	CACGTGTTTTGATCGAAACTTTCGATCTTCGTTTAGGG	38
MS2_101_62	CAAGGTAGCGGAGCGCCTGGCGCCAATTACCGCGACGA	38
MS2_101_63	GCGGCAGTGTACGCCTTCACGAGCGCAATGGTTTGCGT	38
MS2_101_64	CGCGAGTTGTGAGGCTGTCGACCTGGCCTCTGCTAAAG	38

MS2_101_65	CAACACCAAGGTTAAAATTACCCTGGGTGACCTTTTGC	38
MS2_101_66	AGGACTTCGGTCGACGCCCGGTTCGCAACGTTCTGCGG	38
MS2_101_67	CACTTCGATGTAAGTCAAGTTTTGGCTTACAGGGAAGA	38
MS2_101_68	GGCTGTAGCAGGAGCGTGCGTCGAGGGAGAAGCCGAAA	38
MS2_101_69	AGGCAGCCCGATCTATTTTATTATTCTTCGGAACTGTAAA	40
MS2_101_70	ACCCCTTTCTGGAGGTACATATTCATATCAGGCTCCTTAC	40
MS2_101_71	CATTCAGGTCTATACCAACGGATTTGAGCCGGCGTCTGATGAAAGCACCG	50
MS2_101_72	CGAACCATCTACGCTGCCCTGCTGAGCCAGACGCTGGTTGATCGATTGAT	50
MS2_101_73	AGGCGATCGGAGATGGAATCGGATGCAGACGATAAGTCTATCGTCGCAAG	50
MS2_101_74	GGATACGATCGAGATATGAATATAGCTCTGGTGGGAGAAAACTCCACACC	50
MS2_101_75	AAATAGTTCCCATCGTATCGTCTCGCCATCTACGATTCCGTAGTGTGAGC	50
MS2_101_76	CAGAATATCATGGACTCTAGCTCAAATGTGAACCCATTTCCCATTGTGGA	50
MS2_101_77	CTATGGTTCCGGCGTTACCAAAATGGATTTGGGTCGCTTTGACTATTGCC	50
MS2_101_78	CACACGGGGTGCAATCTCACTGGGACATATAATATCGTCCCCGTAGATGC	50
MS2_101_79	AACGTTTTACGAAGATTCGGTTTAAAACCGTAGTAGGCAAGTGCCTCTAG	50
MS2_101_80	CACCACGGTAAAAGTGCGCGCCGCAGCTCTCGCGAAAGAGCCCGGACACG	50
MS2_101_81	GGCGAAGAGATTGTCAACAGGTTTCTTGATGTAAAACGGTTTGACATCGA	50
MS2_101_82	ATACCTCCGACAACTCCCCAACCCCGTAGCCGATTTAATATCAGCATCAG	50
MS2_101_83	GCACCTGGGAGGAGAGCCGTACCCACACCTTATAGAGGCGTGGATCTGAC	50
MS2_101_84	GACTACGTAGTAGTCGGCAGCGAGGTCCGTCCCACCGAAGAACATCGAAG	50
MS2_101_85	AGCCGCCCGTACGGAGTCTTGGTGTATACCGAGACTGCCGTAGGCGGGCT	50
MS2_101_86	CTCGAGCGATACGAGCAAGACGGAAACCCGAGGTACGGGTATCCGCGAGC	50
MS2_101_87	CCACGCTATGTAGCGACCACTGTCGTGCTTTTCGCTGAAGAACTTGCGTT	50
MS2_101_88	CGCACGCCGGCGGACTTCATGCTGTCGGTGATTTCACCTCCAGTATGGAA	50
MS2_101_89	CCTGAGGGAATGTGGGAACCGGCGTTAGCCACTCCGAAGTGCGTATAACG	50
MS2_101_90	TTACGGGGGTCCCTCGGTCAGCTACCGAGGAGAGCTCGCTGGCCCACACT	50
MS2_101_91	GGAGCCGGACCGCTTTCGCACCCGTGCTCTTTCGAGCACACCCACCCCGT	50
MS2_101_92	TCCTCTCTTTAGGGGGAGGTCCCTGGGCCGAAGCCCGCCCACCTTTCGGT	50
MS2_101_1	GGCATTAGCTCGACAGGAAGTTGAGCAGGACCCCGAAAGGGGTCCCACCC	50

Table S2. Oligonucleotide sequences for DNA strands complementary to 18S rRNA and producing the 111 RNA ID. Dumbbell-containing sequences are highlighted in red.

NAME	SEQUENCE (5′ TO 3′)	LENGTH (NT)
18S_111_1	TAATGATCCTTCCGCAGGTTCACCTACGGAAACCTTGT	38
18S_111_2	TACGACTTTTACTTCCTCTAGATAGTCAAGTTCGACCG	38
18S_111_3	TCTTCTCAGCGCTCCGCCAGGGCCGTGGGCCGACCCCG	38
18S_111_4	GCGGGGCCGATCCGAGGGCCTCACTAAACCATCCAATC	38
18S_111_5	GGTAGTAGCGACGGGCGGTGTGTACAAAGGGCAGGGAC	38
18S_111_6	TTAATCAACGCAAGCTTATGACCCGCACTTACTGGGAA	38
18S_111_7	TTCCTCGTTCATGGGGAATAATTGC	25
18S_111_8	AATCCCCGATCCCCATCACGAATGG	25
18S_111_9	GGTTCAACGGTCCTCTTTTGAGGAACAAGTTTTCTTGTGTTACCCGCG	48
18S_111_10	CCTGCCGGCGTCCTCTTTTGAGGAACAAGTTTTCTTGTTAGGGTAGGC	48
18S_111_11	ACACGCTGAGTCCTCTTTTGAGGAACAAGTTTTCTTGTCCAGTCAGTG	48
18S_111_12	TAGCGCGCGTTCCTCTTTTGAGGAACAAGTTTTCTTGTGCAGCCCCGG	48
18S_111_13	ACATCTAAGGTCCTCTTTTGAGGAACAAGTTTTCTTGTGCATCACAGA	48
18S_111_14	CCTGTTATTGTCCTCTTTTGAGGAACAAGTTTTCTTGTCTCAATCTCG	48
18S_111_15	GGTGGCTGAACGCCACTTGTCCCTCTAAGAAGTTGGGG	38
18S_111_16	GACGCCGACCGCTCGGGGGTCGCGTAACTAGTTAGCAT	38
18S_111_17	GCCAGAGTCTCGTTCGTTATCGGAATTAACCAGACAAA	38

18S_111_18	TCGCTCCACCAACTAAGAACGGCCATGCACCACCACCC	38
18S_111_19	ACGGAATCGAGAAAGAGCTATCAATCTGTCAATCCTGT	38
18S_111_20	CCGTGTCCGGGCCGGGTGAGGTTTCCCGTGTTGAGTCA	38
18S_111_21	AATTAAGCCGCAGGCTCCACTCCTG	25
18S_111_22	GTGGTGCCCTTCCGTCAATTCCTTT	25
18S_111_23	AAGTTTCAGCTCCTCTTTTGAGGAACAAGTTTTCTTGTTTTGCAACCA	48
18S_111_24	TACTCCCCCCTCCTCTTTTGAGGAACAAGTTTTCTTGTGGAACCCAAA	48
18S_111_25	GACTTTGGTTTCCTCTTTTGAGGAACAAGTTTTCTTGTTCCCGGAAGC	48
18S_111_26	TGCCCGGCGGTCCTCTTTTGAGGAACAAGTTTTCTTGTGTCATGGGAA	48
18S_111_27	TAACGCCGCCTCCTCTTTTGAGGAACAAGTTTTCTTGTGCATCGCCGG	48
18S_111_28	TCGGCATCGTTCCTCTTTTGAGGAACAAGTTTTCTTGTTTATGGTCGG	48
18S_111_29	AACTACGACGGTATCTGATCGTCTTCGAACCTCCGACT	38
18S_111_30	TTCGTTCTTGATTAATGAAAACATTCTTGGCAAATGCT	38
18S_111_31	TTCGCTCTGGTCCGTCTTGCGCCGGTCCAAGAATTTCA	38
18S_111_32	CCTCTAGCGGCGCAATACGAATGCCCCCGGCCGTCCCT	38
18S_111_33	CTTAATCATGGCCTCAGTTCCGAAAACCAACAAAATAG	38
18S_111_34	AACCGCGGTCCTATTCCATTATTCCTAGCTGCGGTATC	38
18S_111_35	CAGGCGGCTCGGGCCTGCTTTGAAC	25
18S_111_36	ACTCTAATTTTTTCAAAGTAAACGC	25
18S_111_37	TTCGGGCCCCTCCTCTTTTGAGGAACAAGTTTTCTTGTGCGGGACACT	48
18S_111_38	CAGCTAAGAGTCCTCTTTTGAGGAACAAGTTTTCTTGTCATCGAGGGG	48
18S_111_39	GCGCCGAGAGTCCTCTTTTGAGGAACAAGTTTTCTTGTGCAAGGGGCG	48
18S_111_40	GGGACGGGCGTCCTCTTTTGAGGAACAAGTTTTCTTGTGTGGCTCGCC	48
18S_111_41	TCGCGGCGGATCCTCTTTTGAGGAACAAGTTTTCTTGTCCGCCCGCCC	48
18S_111_42	GCTCCCAAGATCCTCTTTTGAGGAACAAGTTTTCTTGTTCCAACTACG	48
18S_111_43	AGCTTTTTAACTGCAGCAACTTTAATATACGCTATTGG	38
18S_111_44	AGCTGGAATTACCGCGGCTGCTGGCACCAGACTTGCCC	38
18S_111_45	TCCAATGGATCCTCGTTAAAGGATTTAAAGTGGACTCA	38
18S_111_46	TTCCAATTACAGGGCCTCGAAAGAGTCCTGTATTGTTA	38
18S_111_47	TTTTTCGTCACTACCTCCCCGGGTCGGGAGTGGGTAAT	38
18S_111_48	TTGCGCGCCTGCTGCCTTCCTTGGATGTGGTAGCCGTT	38
18S_111_49	TCTCAGGCTCCCTCTCCGGAATCGA	25
18S_111_50	ACCCTGATTCCCCGTCACCCGTGGT	25
18S_111_51	CACCATGGTAGGCACGGCGACTACCATCGAAAGTTGATAG	40
18S_111_52	GGCAGACGTTCGAATGGGTCGTCGCCGCCACGGGGGGCGT	40
18S_111_53	GCGATCGGCCCGAGGTTATCTAGAGTCACCAAAGCCGCCG	40
18S_111_54	GCGCCCGCCCCCCGGCCGGGGCCGGAGAGGGGCTGACC	38
18S_111_55	GGGTTGGTTTTGATCTGATAAATGCACGCATCCCCCCC	38
18S_111_56	GCGAAGGGGGTCAGCGCCCGTCGGCATGTATTAGCTCT	38
18S_111_57	AGAATTACCACAGTTATCCAAGTGGGAGAGGAGCGAGC	38
18S_111_58	GACCAAAGGAACCATAACTGATTTAATGAGCCATTCGC	38
18S_111_59	AGTTTCACTGTACCGGCCGTGCGTACTTAGACATGCAT	38
18S_111_60	GGCTTAATCTTTGAGACAAGCATAT	25
18S_111_61	GCTACTGGCAGGATCAACCAGGTA	24

Table S3. Oligonucleotide sequences for DNA strands complementary to 28S rRNA and producing the 11111 RNA ID. Dumbbell-containing sequences are highlighted in red.

NAME	SEQUENCE (5′ TO 3′)	LENGTH (NT)
28S_11111_1	TCGGAACGGCGCTCGCCCATCTCTCAGGACCGACTGAC	38
28S_11111_2	CCATGTTCAACTGCTGTTCACATGGAACCCTTCTCCAC	38
28S_11111_3	TTCGGCCTTCAAAGTTCTCGTTTGAATATTTGCTACTA	38
28S_11111_4	CCACCAAGATCTGCACCTGCGGCGGCTCCACCCGGGCC	38

28S_11111_5	CGCGCCCTAGGCTTCAAGGCTCACCGCAGCGGCCCTCC	38
28S_11111_6	TACTCGTCGCGGCGTAGCGTCCGCGGGGCTCCGGGGGC	38
28S_11111_7	GGGGAGCGGGGCGTGGGCGGGAGGAGGGGAGGAGGCGT	38
28S_11111_8	GGGGGGGGGGGCGGGGGAAGGACCCCACACCCCCGCCG	38
28S_11111_9	CCGCCGCCGCCGCCGCCCTCCGACGCACACCACACGCG	38
28S_11111_10	CGCGCGCGCGCGCCGCCCCCGCCGCTCCCGTCCACTCT	38
28S_11111_11	CGACTGCCGGTCCTCTTTTGAGGAACAAGTTTTCTTGTCGACGGCCGG	48
28S_11111_12	GTATGGGCCCTCCTCTTTTGAGGAACAAGTTTTCTTGTGACGCTCCAG	48
28S_11111_13	CGCCATCCATTCCTCTTTTGAGGAACAAGTTTTCTTGTTTTCAGGGCT	48
28S_11111_14	AGTTGATTCGTCCTCTTTTGAGGAACAAGTTTTCTTGTGCAGGTGAGT	48
28S_11111_15	TGTTACACACTCCTCTTTTGAGGAACAAGTTTTCTTGTTCCTTAGCGG	48
28S_11111_16	ATTCCGACTTTCCTCTTTTGAGGAACAAGTTTTCTTGTCCATGGCCAC	48
28S_11111_17	CGTCCTGCTGTCTATATCAACCAACACCTTTTCTGGGG	38
28S_11111_18	TCTGATGAGCGTCGGCATCGGGCGCCTTAACCCGGCGT	38
28S_11111_19	TCGGTTCATCCCGCAGCGCCAGTTCTGCTTACCAAAAG	38
28S_11111_20	TGGCCCACTAGGCACTCGCATTCCACGCCCGGCTCCAC	38
28S_11111_21	GCCAGCGAGCCGGGCTTCTTACCCATTTAAAGTTTGAG	38
28S_11111_22	AATAGGTTGAGATCGTTTCGGCCCCAAGACCTCTAATC	38
28S_11111_23	ATTCGCTTTACCGGATAAAACTGCG	25
28S_11111_24	TGGCGGGGGTGCGTCGGGTCTGCGA	25
28S_11111_25	GAGCGCCAGCTCCTCTTTTGAGGAACAAGTTTTCTTGTTATCCTGAGG	48
28S_11111_26	GAAACTTCGGTCCTCTTTTGAGGAACAAGTTTTCTTGTAGGGAACCAG	48
28S_11111_27	CTACTAGATGTCCTCTTTTGAGGAACAAGTTTTCTTGTGTTCGATTAG	48
28S_11111_28	TCTTTCGCCCTCCTCTTTTGAGGAACAAGTTTTCTTGTCTATACCCAG	48
28S_11111_29	GTCGGACGACTCCTCTTTTGAGGAACAAGTTTTCTTGTCGATTTGCAC	48
28S_11111_30	GTCAGGACCGTCCTCTTTTGAGGAACAAGTTTTCTTGTCTACGGACCT	48
28S_11111_31	CCACCAGAGTTTCCTCTGGCTTCGCCCTGCCCAGGCAT	38
28S_11111_32	AGTTCACCATCTTTCGGGTCCTAACACGTGCGCTCGTG	38
28S_11111_33	CTCCACCTCCCCGGCGCGGCGGGCGAGACGGGCCGGTG	38
28S_11111_34	GTGCGCCCTCGGCGGACTGGAGAGGCCTCGGGATCCCA	38
28S_11111_35	CCTCGGCCGGCGAGCGCGCCGGCCTTCACCTTCATTGC	38
28S_11111_36	GCCACGGCGGCTTTCGTGCGAGCCCCCGACTCGCGCAC	38
28S_11111_37	GTGTTAGACTCCTTGGTCCGTGTTT	25
28S_11111_38	CAAGACGGGTCGGGTGGGTAGCCGA	25
28S_11111_39	CGTCGCCGCCTCCTCTTTTGAGGAACAAGTTTTCTTGTGACCCCGTGC	48
28S_11111_40	GCTCGCTCCGTCCTCTTTTGAGGAACAAGTTTTCTTGTCCGTCCCCCT	48
28S_11111_41	CTTCGGGGGATCCTCTTTTGAGGAACAAGTTTTCTTGTCGCGCGCGTG	48
28S_11111_42	GCCCCGAGAGTCCTCTTTTGAGGAACAAGTTTTCTTGTAACCTCCCCC	48
28S_11111_43	GGGCCCGACGTCCTCTTTTGAGGAACAAGTTTTCTTGTGCGCGACCCG	48
28S_11111_44	CCCGGGGCGCTCCTCTTTTGAGGAACAAGTTTTCTTGTACTGGGGACA	48
28S_11111_45	GTCCGCCCCGCCCCCCGACCCGCGCGCGGCACCCCCCC	38
28S_11111_46	CGTCGCCGGGGCGGGGGCGCGGGGAGGAGGGGTGGGAG	38
28S_11111_47	AGCGGTCGCGCCGTGGGAGGGGTGGCCCGGCCCCCCCA	38
28S_11111_48	CGAGGAGACGCCGGCGCGCCCCCGCGGGGGAGACCCCC	38
28S_11111_49	CTCGCGGGGGATTCCCCGCGGGGGTGGGCGCCGGGAGG	38
28S_11111_50	GGGGAGAGCGCGGCGACGGGTCTCGCTCCCTCGGCCCC	38
28S_11111_51	GGGATTCGGCGAGTGCTGCTGCCGG	25
28S_11111_52	GGGGGCTGTAACACTCGGGGGGGGT	25
28S_11111_53	TTCGGTCCCGTCCTCTTTTGAGGAACAAGTTTTCTTGTCCGCCGCCGC	48
28S_11111_54	CGCCGCCGCCTCCTCTTTTGAGGAACAAGTTTTCTTGTACCGCCGCCG	48
28S_11111_55	CCGCCGCCGCTCCTCTTTTGAGGAACAAGTTTTCTTGTCCCGACCCGC	48
28S_11111_56	GCGCCCTCCCTCCTCTTTTGAGGAACAAGTTTTCTTGTGAGGGAGGAC	48
28S_11111_57	GCGGGGCCGGTCCTCTTTTGAGGAACAAGTTTTCTTGTGGGGCGGAGA	48
28S_11111_58	CGGGGGAGGATCCTCTTTTGAGGAACAAGTTTTCTTGTGGAGGACGGA	48
28S_11111_59	CGGACGGACGGACGGGGCCCCCCGAGCCACCTTCCCCG	38
28S_11111_60	CCGGGCCTTCCCAGCCGTCCCGGAGCCGGTCGCGGCGC	38
28S_11111_61	ACCGCCGCGGTGGAAATGCGCCCGGCGGCGGCCGGTCG	38

28S_11111_62	CCGGTCGGGGGACGGTCCCCCGCCGACCCCACCCCCGG	38
28S_11111_63	CCCCGCCCGCCCACCCCCGCACCCGCCGGAGCCCGCCC	38
28S_11111_64	CCTCCGGGGAGGAGGAGGAGGGGCGGCGGGGGAAGGGA	38
28S_11111_65	GGGCGGGTGGAGGGGTCGGGAGGAA	25
28S_11111_66	CGGGGGGCGGGAAAGATCCGCCGGG	25
28S_11111_67	CCGCCGACACTCCTCTTTTGAGGAACAAGTTTTCTTGTGGCCGGACCC	48
28S_11111_68	GCCGCCGGGTTCCTCTTTTGAGGAACAAGTTTTCTTGTTGAATCCTCC	48
28S_11111_69	GGGCGGACTGTCCTCTTTTGAGGAACAAGTTTTCTTGTCGCGGACCCC	48
28S_11111_70	ACCCGTTTACTCCTCTTTTGAGGAACAAGTTTTCTTGTCTCTTAACGG	48
28S_11111_71	TTTCACGCCCTCCTCTTTTGAGGAACAAGTTTTCTTGTTCTTGAACTC	48
28S_11111_72	TCTCTTCAAATCCTCTTTTGAGGAACAAGTTTTCTTGTGTTCTTTTCA	48
28S_11111_73	ACTTTCCCTTACGGTACTTGTTGACTATCGGTCTCGTG	38
28S_11111_74	CCGGTATTTAGCCTTAGATGGAGTTTACCACCCGCTTT	38
28S_11111_75	GGGCTGCATTCCCAAGCAACCCGACTCCGGGAAGACCC	38
28S_11111_76	GGGCCCGGCGCGCCGGGGGCCGCTACCGGCCTCACACC	38
28S_11111_77	GTCCACGGGCTGGGCCTCGATCAGAAGGACTTGGGCCC	38
28S_11111_78	CCCACGAGCGGCGCCGGGGAGCGGGTCTTCCGTACGCC	38
28S_11111_79	ACATGTCCCGCGCCCCGCCGCGGGGCGGGGATTCGGCG	38
28S_11111_80	CTGGGCTCTTCCCTGTTCACTCGCCGTTACTGAGGGAA	38
28S_11111_81	TCCTGGTTAGTTTCTTCTCCTCCGCTGACTAATATGCT	38
28S_11111_82	TAAATTCAGCGGGTCGCCACGTCTGATCTGAGGTCGCG	38
28S_11111_83	GACAAACCCTTGTGTCGAGGGCTGACTTTCAATAGATC	38
28S_11111_84	GCAGCGAGGGAGCTGCTCTGCTACGTACGAAACCCCGA	38
28S_11111_85	CCCAGAAGCAGGTCGTCTACGAATGGTTTAGCGCCAGG	38
28S_11111_86	TTCCCCACGAACGTGCGGTGCGTGACGGGCGAGGGGGC	38
28S_11111_87	GGCCGCCTCTCCGGCCGCGCCCCGTTTCCCAGGACGAA	38
28S_11111_88	GGGCACTCCGCACCGGACCCCGGTCCCGGCGCGCGGCG	38
28S_11111_89	GGGCACGCGCCCTCCCGCGCGCGCGGGGCGCGTGGAGG	38
28S_11111_90	GGGGGGGCGGCCCGCCGGCGGGGACAGGCGGGGGACCG	38
28S_11111_91	GCTATCCGAGGCCAACCGAGGCTCCGCGGCGCTGCCGT	38
28S_11111_92	ATCGTTCCGCCTGGGCGGGATTCTGACTTAGAGGCGTT	38
28S_11111_93	CAGTCATAATCCCACAGATGGTAGCTTCGCCCCATTGG	38
28S_11111_94	CTCCTCAGCCAAGCACATACACCAAATGTCTGAACCTG	38
28S_11111_95	CGGTTCCTCTCGTACTGAGCAGGATTACCATGGCAACA	38
28S_11111_96	ACACATCATCAGTAGGGTAAAACTAACCTGTCTCACGA	38
28S_11111_97	CGGTCTAAACCCAGCTCACGTTCCCTATTAGTGGGTGA	38
28S_11111_98	ACAATCCAACGCTTGGTGAATTCTGCTTCACAATGATA	38
28S_11111_99	GGAAGAGCCGACATCGAAGGATCAAAAAGCGACGTCGC	38
28S_11111_100	TATGAACGCTTGGCCGCCACAAGCCAGTTATCCCTGTG	38
28S_11111_101	GTAACTTTTCTGACACCTCCTGCTTAAAACCCAAAAGG	38
28S_11111_102	TCAGAAGGATCGTGAGGCCCCGCTTTCACGGTCTGTAT	38
28S_11111_103	TCGTACTGAAAATCAAGATCAAGCGAGCTTTTGCCCTT	38
28S_11111_104	CTGCTCCACGGGAGGTTTCTGTCCTCCCTGAGCTCGCC	38
28S_11111_105	TTAGGACACCTGCGTTACCGTTTGACAGGTGTACCGCC	38
28S_11111_106	CCAGTCAAACTCCCCACCTGGCACTGTCCCCGGAGCGG	38
28S_11111_107	GTCGCGCCCGGCCGGCGCGCGGCCGGGCGCTTGGCGCC	38
28S_11111_108	AGAAGCGAGAGCCCCTCGGGGCTCGCCCCCCCGCCTCA	38
28S_11111_109	CCGGGTCAGTGAAAAAACGATCAGAGTAGTGGTATTTC	38
28S_11111_110	ACCGGCGGCCCGCAGGGCCGGCGGACCCCGCCCCGGGC	38
28S_11111_111	CCCTCGCGGGGACACCGGGGGGGCGCCGGGGGCCTCCC	38
28S_11111_112	ACTTATTCTACACCTCTCATGTCTCTTCACCGTGCCAG	38
28S_11111_113	ACTAGAGTCAAGCTCAACAGGGTCTTCTTTCCCCGCTG	38
28S_11111_114	ATTCCGCCAAGCCCGTTCCCTTGGCTGTGGTTTCGCTG	38
28S_11111_115	GATAGTAGGTAGGGACAGTGGGAATCTCGTTCATCCAT	38
28S_11111_116	TCATGCGCGTCACTAATTAGATGACGAGGCATTTGGCT	38
28S_11111_117	ACCTTAAGAGAGTCATAGTTACTCCCGCCGTTTACCCG	38
28S_11111_118	CGCTTCATTGAATTTCTTCACTTTGACATTCAGAGCAC	38

28S_11111_119	TGGGCAGAAATCACATCGCGTCAACACCCGCCGCGGGC	38
28S_11111_120	CTTCGCGATGCTTTGTTTTAATTAAACAGTCGGATTCC	38
28S_11111_121	CCTGGTCCGCACCAGTTCTAAGTCGGCTGCTAGGCGCC	38
28S_11111_122	GGCCGAGGCGAGGCGCCGCGCGGAACCGCGGCCCCGGG	38
28S_11111_123	GGCGGACCCGGCGGGGGGGACCGGCCCGCGGCCCCTCC	38
28S_11111_124	GCCGCCTGCCGCCGCCGCCGCCGCGCGCCGAGGAGGAG	38
28S_11111_125	GGGGGAACGGGGGGCGGACGGGGCCGGGGGGGTAGGGC	38
28S_11111_126	GGGGGGACGAACCGCCCCGCCCCGCCGCCCGCCGACCG	38
28S_11111_127	CCGCCGCCCGACCGCTCCCCGCCCCCAGCGGACGCGCG	38
28S_11111_128	CGCGACGAGACGTGGGGTGGGGGGGGGGCGCGCCGGC	38
28S_11111_129	GCCCGCCGGGCTCCCCGGGGGCGGCCGCGACGCCCGCC	38
28S_11111_130	GCAGCTGGGGCGATCCACGGGAAGGGCCCGGCTCGCGT	38
28S_11111_131	CCAGAGTCGCCGCCGCCGCCGGCCCCCCGGGTGCCCGG	38
28S_11111_132	GCCCCCCTCGCGGGGGACCGTGCCCCCGCCGCCGGGGC	38
28S_11111_133	CCCGCGGCGGGCCGCCGCCGGCCCCTGCCGCCCCGACC	38
28S_11111_134	CTTCTCCCCCCGCCGCCGCCCCCACGCGGCGCTCCCCC	38
28S_11111_135	GGGGAGGGGGGAGGACGGGGAGCGGGGGAGAGAGAGAG	38
28S_11111_136	AGAGAGGGCGCGGGGCGGGGAGGGAGCGAGCGGCGCGC	38
28S_11111_137	GCGGGGTGGGGCGGGGGAGGGCCGCGAGGGGGGTGCCC	38
28S_11111_138	CGGGCGTGGGGGGGGCGGCGGCGCCTCGTCCAGCCGCG	38
28S_11111_139	GCGCGCGCCCAGCCCCGCTTCGCGCCCCAGCCCGACCG	38
28S_11111_140	ACCCAGCCCTTAGAGCCAATCCTTATCCCGAAGTTACG	38
28S_11111_141	GATCCGGCTTGCCGACTTCCCTTACCTACATTGTTCCA	38
28S_11111_142	ACATGCCAGAGGCTGTTCACCTTGGAGACCTGCTGCGG	38
28S_11111_143	ATATGGGTACGGCCCGGCGCGAGATTTACACCCTCTCC	38
28S_11111_144	CCCGGATTTTCAAGGGCCAGCGAGAGCTCACCGGACGC	38
28S_11111_145	CGCCGGAACCGCGACGCTTTCCAAGGCACGGGCCCCTC	38
28S_11111_146	TCTCGGGGCGAACCCATTCCAGGGCGCCCTGCCCTTCA	38
28S_11111_147	CAAAGAAAAGAGAACTCTCCCCGGGGCTCCCGCCGGCT	38
28S_11111_148	TCTCCGGGATCGGTCGCGTTACCGCACTGGACGCCTCG	38
28S_11111_149	CGGCGCCCATCTCCGCCACTCCGGATTCGGGGATCTGA	38
28S_11111_150	ACCCGACTCCCTTTCGATCGGCCGAG	26
28S_11111_151	GGCAACGGAGGCCATCGCCCGTCCCT	26

10. References

1. Bošković, F., Keyser, U.F. Nanopore microscope identifies RNA isoforms with structural colours. *Nat. Chem.* **14**, 1258–1264 (2022).
